# Upcycling of Pomegranate By‐Products: Pomegranate Juice Enrichment With Phenolics‐Rich Pomegranate By‐Product Extracts Obtained by Green Extraction Methods

**DOI:** 10.1002/fsn3.70250

**Published:** 2025-05-29

**Authors:** Merve Aydin, Ismail Tontul, Selman Turker

**Affiliations:** ^1^ Department of Food Engineering, Faculty of Engineering Necmettin Erbakan University Konya Türkiye

**Keywords:** enzymatic extraction, homogenization, phenolic compounds, pomegranate juice, storage, upcycling

## Abstract

The objective of this study was to produce high‐added‐value and upcycled pomegranate juices (PJs) enriched with extracts obtained from pomegranate by‐products using innovative green extraction methods. The physicochemical properties of PJs stored for 60 days at 4°C and 25°C were analyzed. The incorporation of extracts into the juice resulted in a notable enhancement in the bioactive content and antioxidant activity of the samples when compared to the control. Conversely, the addition of extracts into juices resulted in a reduction in L* value, appearance and color values, and elevated turbidity. The sensory evaluation indicated that the PJ containing extract obtained from enzymatic and solvent‐assisted extraction exhibited the most sensory appreciation in terms of odor, taste, aftertaste, and general acceptability. The prolongation of storage time and elevation of temperature resulted in a decline in a*, b*, and hue angle values, accompanied by a reduction in phenolics, total monomeric anthocyanin, and antioxidant activity. All sensory properties of the samples decreased during the storage period. To maintain the quality of enriched PJs, they should be stored at 4°C. Consequently, the production of upcycled PJs with a high concentration of bioactive components and a sensory profile that was acceptable was achieved. Furthermore, enrichment of the PJ with H extract, with its low addition rate and brief extraction time, was an optimum option in terms of chemical, physical, and sensory properties and industry.

## Graphical abstract

The objective of this study was to produce high‐added‐value and upcycled pomegranate juices (PJs) enriched with extracts obtained from pomegranate by‐products using innovative green extraction methods. The incorporation of extracts into the juice resulted in a notable enhancement in the bioactive content and antioxidant activity of the samples when compared to the control. The sensory evaluation indicated that the PJ containing extract obtained from enzymatic and solvent‐assisted extraction had a good sensorial acceptability.

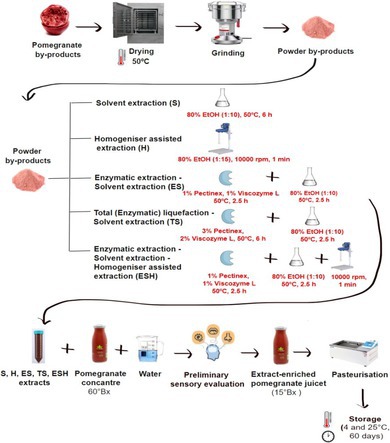

## 1 Introduction

In recent years, there has been a notable increase in consumer demand for value‐added food products. Similarly, the beverage sector has concentrated on enhancing its products with bioactive components in order to align itself with this trend. In order to achieve this, there has been a focus on the recovery of bioactive components from fruit and vegetable by‐products (Aydin et al. 2024). The process of converting the pomegranate ( *Punica granatum* ), renowned for its palatable flavor and beneficial physiological effects, into products such as pomegranate juice (PJ), jelly, syrup, jam, and colorant generates a multitude of by‐products (40%–50% of the total fruit weight). These by‐products comprise important bioactive compounds, including hydrolyzable tannins, flavonoids, anthocyanins, phenolic acids, phytosterols, and polyunsaturated fatty acids (Andrade et al. 2019). A substantial body of evidence from scientific studies indicates that phenolic compounds found in high amounts in pomegranate and its by‐products possess a range of beneficial properties, including antioxidant, antibacterial, anti‐inflammatory, anti‐allergic, anticancer, anti‐diabetic, and cardiovascular disease preventive effects (Hegazi et al. 2021). These bioactive properties are primarily attributed to the high ellagitannin content in pomegranate (Seeram et al. 2005; Eroglu Ozkan et al. 2021).

The inability to extract polyphenolic components from the plant matrix using a solvent, due to their bound and/or large molecular structure, results in a reduction in the anticipated health benefits and bioavailability of these components. Conventional techniques for the recovery of these components are associated with significant drawbacks, including prolonged processing times, substantial financial expenditures, considerable environmental impact, and the deterioration of heat‐sensitive compounds (Trigo, Alexandre, Silva, et al. 2020). As a matter of fact, green extraction methods provide an alternative to traditional extraction methods, given that they reduce the use of toxic solvents, and are fast, energy‐saving, high‐efficiency, economical, eco‐friendly, and sustainable (Aydin et al. 2024). Enzyme‐assisted extraction represents a time‐saving, eco‐friendly, and biologically promising process for the extraction of phenolic compounds with high stability, yield, and antioxidant activity (Mushtaq et al. 2016; Aydin et al. 2024). In our previous study, we used enzymatic liquefaction (2% Viscozyme L + 3% Pectinex) and ultrafiltration combination processes as green methods for the extraction of phenolic compounds from pomegranate by‐products (Aydin et al. 2025). Another green method is homogenizer‐assisted extraction, which is frequently employed due to its sustainability, simplicity, efficacy, and cost‐effectiveness (Eyiz et al. 2020).

The incorporation of phenolic compounds derived from fruit and vegetable by‐products into fruit and vegetable juices has emerged as a notable trend in recent times. In numerous studies, the phenolic extracts derived from diverse medicinal aromatic plants (thyme, sage) and fruit and vegetable by‐products (beet leaves, pomegranate peel) through various extraction techniques have been employed in the fabrication of value‐added beverages (apple, pineapple, orange, and carrot juices) (Fernandez et al. 2020; Trigo, Alexandre, Oliveira, et al. 2020; Maleš et al. 2023). This has resulted in an enhancement of the functional properties of the beverages, primarily due to an increase in the phenolic concentration and antioxidant potential of fruit and vegetable juices. Nevertheless, the position of these functional beverages in the food market has yet to reach the desired level. In the present study, the PJ was selected due to its higher phenolic content compared to other fruit juices, and its enrichment of this content with its own by‐products without the addition of any external additives. Furthermore, its unique taste and color could suppress the added extract effectively, so it was preferred over other fruit juices. To the best of our knowledge, our previous publication is the only study on the extraction of phenolic compounds from pomegranate by‐products by enzymatic liquefaction (Aydin et al. 2025). However, no study was found on the use of homogenizer‐assisted and enzymatic‐assisted extractions to obtain the phenolic extract from the pomegranate by‐products and its use in the upcycling of the PJs. There is a lack of research on the separate and combined incorporation of phenolic extracts into the PJ using green methods.

A novel functional product that reduces food waste obtained by reintroducing bioactive compounds recovered from by‐products into the food production line as raw materials is defined as “upcycled food”. Furthermore, the contributions of these evaluated by‐products to reduce environmental pollution and increase the profits of producers also ensure their sustainability. Additionally, the innovative methods used in the extraction of bioactive compounds significantly reduce carbon‐water footprints compared to traditional methods, thereby enhancing the sustainability of the process (Aschemann‐Witzel et al. 2023). Examples of upcycled food in a novel use sense include the conversion of spent coffee into flour, the processing of olive leaves into crackers, the production of a snack from fish skin, and the utilization of grass, pine tree needles, or bark as a source of protein (Aschemann‐Witzel and Peschel 2019; Aschemann‐Witzel et al. 2023).

The objective of this study is to produce an upcycled, sustainable, and value‐added product with high antioxidant capacity, functional and sensory enhancement by adding antioxidant‐rich phenolic compounds from pomegranate by‐products to PJ using green and innovative methods, including enzymatic extraction, total liquefaction, and homogenizer‐assisted extraction. Additionally, the effect of storage temperature (4°C and 25°C) and time (60 days) on some quality characteristics of these enriched PJs was investigated.

## 2 Materials and Methods

### 2.1 Materials

The pomegranates used in the study were purchased from the local market. The pomegranate concentrates (60°Bx) were provided by Döhler Ltd. Co. (Karaman, Türkiye). The commercial enzymes (Pectinex Ultra SP‐L and Viscozyme‐L) were sourced from Novozymes (Bagsvaerd, Denmark). All chemicals and solvents used in the analyses were of analytical grade and obtained from Merck (Germany).

### 2.2 Extraction of Pomegranate By‐Products

Following the manual squeezing of the pomegranates, the peels, pulps, and seeds (by‐products) were separated. These were then subjected to drying (50°C, ≤ 3% moisture) and grinding (Altunkaya et al. 2013). They were subsequently extracted using five different methods.

#### 2.2.1 Solvent Extraction (S)

An 80% ethanol solution in a ratio of 1:10 (g:mL) was combined with powdered pomegranate by‐products and incubated in a water bath at 50°C with agitation at 100 rpm for 6 h. Then, the mixture was subjected to centrifugation (10,000 rpm, 10 min), filtration (150 μm) and rotary evaporation (50°C). Thereafter, the extract was stored at −20°C (Bosso et al. 2016).

#### 2.2.2 Homogenizer Assisted Extraction (H)

An 80% ethanol solution in a ratio of 1:15 (g:mL) was combined with powdered pomegranate by‐products and homogenized using an ultraturrax (IKA T25 digital, Germany) at 10,000 rpm for 1 min (Eyiz et al. 2020). Subsequently, the concentrated extract was obtained and stored under the same conditions as the solvent extraction.

#### 2.2.3 Enzymatic Extraction—Solvent Extraction (ES)

The pomegranate by‐products were rehydrated with a buffer solution (pH 5) in a ratio of 1:6 (g/mL) for 1 h. Subsequently, Pectinex (1%, v/w) and Viscozyme L enzymes (1%, v/w) were added to the mixture in accordance with the initial weight of the powdered sample. Then, the mixture was incubated in a water bath at 50°C with agitation at 100 rpm for 2.5 h. Thereafter, the reaction was terminated by heating the mixture to 90°C for 3 min (Maier et al. 2008; Mushtaq et al. 2016). Finally, the solvent extraction procedure was applied as previously described.

#### 2.2.4 Total (Enzymatic) Liquefaction—Solvent Extraction (TS)

This extraction method is analogous to Method 2.2.3, but with an augmented enzyme concentration and an extended enzymatic process duration. In order to achieve this, Pectinex was employed at a concentration of 3%, and Viscozyme L was used at a concentration of 2%. The enzymatic process was conducted for a period of 6 h. All other temperatures, times, and ratios were maintained at the same levels as those employed in the enzymatic process (Method 3).

#### 2.2.5 Enzymatic Extraction—Solvent Extraction—Homogenizer Assisted Extraction (ESH)

The enzymatic treatment was conducted in accordance with method 2.2.3, and the resulting mixture was subsequently homogenized using an ultraturrax at 10,000 rpm for 1 min. Subsequently, solvent extraction was employed.

### 2.3 Enrichment of Pomegranate Juice (PJs) With Pomegranate By‐Product Extracts

The pomegranate concentrate was diluted to a concentration of 15°Bx with distilled water and employed as a control sample. The extracts obtained through different extraction methods were added to the pomegranate concentrate in accordance with the preliminary sensory evaluation results. Accordingly, the pomegranate concentrates were supplemented with 6%–10% (g/100 mL) extracts (S: 10%, ESH: 8%, ES: 7%, TS: 7% and H: 6%) and then they were adjusted to 15°Bx with distilled water. Subsequently, the control and extract‐enriched PJ samples were subjected to a 2‐min pasteurization process at 95°C.

### 2.4 Storage

The PJs were stored at two different temperatures (4°C and 25°C) over a 60‐day period (Salgado et al. 2012; Altunkaya et al. 2013). The samples were analyzed at 15‐day intervals, with two parallel sets of samples stored at each time point for subsequent analysis.

### 2.5 Total Soluble Solid Content (Brix)

The soluble solid content (°Bx) of the samples was determined using a refractometer (Atago‐Pal Alpha, Japan).

### 2.6 Turbidity

Once the PJ samples had reached room temperature, their turbidity was quantified utilizing a turbidimeter (Hach 2100Q, Loveland, CO, USA) as Nephelometric Turbidity Units (NTU) (Oziyci et al. 2013).

### 2.7 Color

The color values of the samples were determined utilizing the Minolta CR‐400 device (Konica Minolta, Osaka, Japan). The device was calibrated in accordance with the CIE Standard Illuminant C. The samples were placed into glass petri dishes and the L* [0 black, 100 white], a* [+red, −green] and b* [+yellow, −blue] values were read from different locations on a white background. The hue angle and saturation values of the samples were calculated using Equations (1) and (2), respectively (Gómez‐Plaza et al. 2006).

(1) $$\mathrm{Hue}\;\text{angle}\;\left(H^{{}^{\circ}}\right){=\tan}^{-1}\left(b^{*}/a^{*}\right)$$

(2) $$\text{Chroma}\;\left(C^{*}\right)=\sqrt{a^{*2}+b^{*2}}$$

### 2.8 Total Phenolic Compound Content

The total phenolic content of the samples was analyzed using the method described by Eyiz et al. (2020), with the results expressed as gallic acid equivalents (mg GAE/mL).

### 2.9 Hydrolyzable Tannin Content

The hydrolyzable tannin content was determined in accordance with the methodology employed by Saffarzadeh‐Matin and Khosrowshahi (2017). The resulting data are expressed as mg tannic acid equivalent (TAE)/mL.

### 2.10 Total Flavonoid Compound Content

The total flavonoid content of the samples was determined in accordance with the methodology outlined by Eyiz et al. (2020). The total flavonoid content of the samples was calculated in milligrams of rutin equivalent (RE) per milliliter.

### 2.11 Total Monomeric Anthocyanin Content

The total monomeric anthocyanin content was determined using a method described by Eyiz et al. (2020), with the results expressed in milligrams of cyanidin‐3‐glucoside (C3G) per liter.

### 2.12 Antioxidant Activity

The antioxidant activity of the samples was evaluated through the analysis of their DPPH (2,2‐diphenyl‐1‐picrylhydrazyl) radical scavenging activity, in accordance with the methodology previously established by Tontul and Topuz (2017). The results were calculated as milligrams of Trolox equivalent antioxidant activity (TEAA) per milliliter.

### 2.13 Sensory Analysis

The sensory analyses of the samples were conducted by a panel of 25 trained and experienced panelists, aged between 25 and 55, at the Department of Food Engineering, Necmettin Erbakan University. The panelists were selected based on their expertise in the field of sensory analysis and their ability to provide objective and reliable feedback. The samples comprising different extracts were randomly assigned alphanumeric codes and served in a randomized order at the same time. The panelists were instructed to use water and salted stick pretzels to cleanse their palates between the evaluated samples. The appearance, color, odor, viscosity, taste, aftertaste, and overall acceptability of the samples were evaluated on a 9‐point hedonic scale (1: strongly dislike, 5: neither like nor dislike, 9: strongly like) (Altunkaya et al. 2013).

### 2.14 Statistical Analyses

The study was designed with three independent variables (PJ variety, storage temperature and time) and eight dependent variables (turbidity, color, total phenolic compound, hydrolyzable tannin, total flavonoid compound and total monomeric anthocyanin contents, antioxidant activity and sensory). The analyses were conducted with two replicates, and the resulting data were presented with mean values ± standard deviations. The data were statistically subjected to variance analysis using the SPSS v.21 software (IBM Corp., Armonk, New York, USA). The univariate general linear model method was used for the data analysis, and each dependent variable was analyzed individually. The significant differences between the independent variables were compared with Duncan's Multiple Comparison Test (*p* ≤ 0.05). The effects of independent variables on the analyses were evaluated with two and three interactions (*p* < 0.01 and *p* < 0.05). The figures were generated using GraphPad Prism v. 8.0.1 (GraphPad Software Inc., La Jolla, CA, USA).

## 3 Results and Discussion

### 3.1 Total Soluble Solid Content

A slight yet statistically significant difference was observed in the total soluble solid content of the pomegranate juice (PJ) samples. Duncan Multiple Comparison Test results showed that the PJ containing extract obtained from solvent extraction (SPJ) exhibited the highest brix value (15.21), while the control sample demonstrated the lowest brix value (15.00) among the PJ samples (Table 1). As the temperature and duration of storage were increased, a corresponding increase in brix degree was observed. However, since the slope of the temperature and storage period interaction curve at 25°C is higher compared to 4°C, the reaction that causes the brix increase is faster at this temperature (*p* ≤ 0.01) (Figure S1). In a previous study, a slight increase in the brix content of pasteurized orange juice with increasing storage temperature (20°C, 28°C, 35°C and 42°C) was explained by the formation of soluble degradation products as a function of time and temperature (Wibowo et al. 2015). According to Wang et al. (2022), the brix content of cloudy apple juice stored at 0°C and 20°C for 90 days increased on the 15th day at 0°C and decreased on the 60th day. When stored at 20°C, there was an increase on the 15th day and a decrease on the subsequent days. The researchers explained that this result was due to starch hydrolysis during the first 15 days. In another study, Mgaya‐Kilima et al. (2015) observed an increase in the brix degree of mixtures containing different proportions of mango juice and hibiscus extract during 6 months of storage. The observed increase in brix during storage can be attributed to the hydrolysis of polysaccharides and partial dehydration (Pareek et al. 2011). It is hypothesized that the extracts added to PJ contain sucrose and some oligosaccharides. Thus, the elevation in temperature and the prolongation of storage may have facilitated the hydrolysis of these compounds. Furthermore, it is possible that added phenolics may form insoluble structures with other compounds during storage. It can therefore be concluded that phenolic compounds do not have a significant effect on brix content.

**TABLE 1:** Total soluble solid content (brix) of pomegranate juice (PJ) samples according to Duncan Multiple Comparison Test results.

Variation sources	*n*	Total soluble solids content (Brix)
PJ sample		
Control	20	15.00 ± 0.03^e^
SPJ	20	15.21 ± 0.03^a^
HPJ	20	15.05 ± 0.03^d^
ESPJ	20	15.09 ± 0.03^c^
TSPJ	20	15.10 ± 0.04^c^
ESHPJ	20	15.17 ± 0.04^b^
Temperature (°C)		
4	60	15.07 ± 0.02^b^
25	60	15.13 ± 0.03^a^
Storage time (Day)		
0	24	14.89 ± 0.02^e^
15	24	14.99 ± 0.02^d^
30	24	15.12 ± 0.02^c^
45	24	15.23 ± 0.02^b^
60	24	15.27 ± 0.02^a^

*Note:* The data indicates a statistically significant difference between the means represented by different letters within the same column (*p* ≤ 0.05). SPJ: The PJ containing extract obtained from solvent extraction. HPJ: The PJ containing the extract obtained from homogenizer‐assisted extraction. ESPJ: The PJ containing extract obtained from enzymatic and solvent‐assisted extraction. TSPJ: The PJ containing the extract obtained from total liquefaction and solvent‐assisted extraction. ESHPJ: The PJ containing extract obtained from enzymatic, solvent, and homogenizer‐assisted extraction.

### 3.2 Turbidity

**FIGURE 1:**
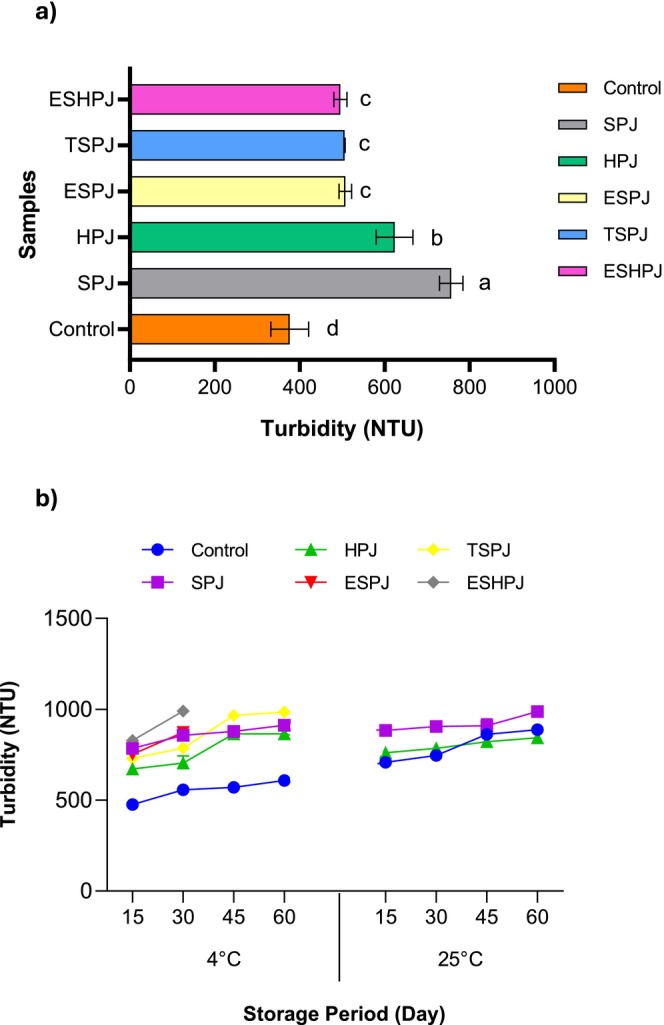
Turbidity results of pomegranate juice (PJ) samples (a), interaction between temperature, storage time, and pomegranate juice variety on the turbidity values of the samples (b). There is a statistical difference between the mean values shown with different letters in the same color (*p* ≤ 0.05). ESHPJ: The PJ containing extract obtained from enzymatic, solvent, and homogenizer‐assisted extraction. ESPJ: The PJ containing extract obtained from enzymatic and solvent‐assisted extraction. HPJ: The PJ containing the extract obtained from homogenizer‐assisted extraction. SPJ: The PJ containing extract obtained from solvent extraction. TSPJ: The PJ containing the extract obtained from total liquefaction and solvent‐assisted extraction.

As some PJ samples exhibited turbidity values exceeding the measurement limit during storage, a statistical analysis was only conducted on Day 0, with the results presented in Figure 1a. The highest turbidity value was observed in the SPJ sample, which exhibited the highest concentration of extract, while the lowest turbidity value was observed in the control. The addition of extract to PJ resulted in an increase in turbidity, as compared to the control sample. Moreover, the turbidity values of the samples containing an enzymatically treated extract were found to be statistically indistinguishable from one another (*p* > 0.05). It is anticipated that the extracts obtained in the study will comprise not only phenolic compounds but also solvents, enzyme residues, and polymeric structures (carbohydrate and protein). Phenolic components exert a considerable influence on the formation of haze. The formation of high molecular weight structures is a consequence of the oxidation of catechins and procyanidins, which are primarily responsible for the observed turbidity. The polymerization of these structures results in the generation of large particles, which contribute to cloudiness and subsequent sedimentation over time (Tetik et al. 2007).

Figure 1b illustrates the interaction between temperature, storage time, and PJ variety on the turbidity values of the samples. The increase in turbidity value of the control sample stored at 25°C was significantly greater than that observed for the same sample stored at 4°C. In the storage of enzyme‐treated samples, it was observed that the rate of turbidity increase at 4°C was in the order of ESHPJ (the PJ containing extract obtained from enzymatic, solvent and homogenizer‐assisted extraction) > ESPJ (the PJ containing extract obtained from enzymatic and solvent‐assisted extraction) > TSPJ (the PJ containing the extract obtained from total liquefaction and solvent‐assisted extraction). Furthermore, the turbidity values of these samples surpassed the measurement limit on the 15th day of storage at 25°C. The formation of turbidity at elevated temperatures is attributed to the higher molecular mobility and/or reactivity of the molecules responsible for turbidity, relative to those observed at lower temperatures (Muche et al. 2018). It is possible that the enzyme residue of the samples may be involved in the formation of turbidity because of polyphenol‐protein interaction (Wang et al. 2016). Moreover, the total phenolic content of the PJs may influence the turbidity of the samples during storage, potentially leading to the formation of haze.

### 3.3 Color

As illustrated in Table 2, the highest L* and hue angle values according to the Duncan Multiple Comparison Test were observed in the control sample, while the highest a* value was found in the ESHPJ sample. Moreover, the highest b* and chroma values were exhibited by both the SPJ and ESHPJ samples. In comparison to the control sample, the addition of the extract to the PJ resulted in a reduction in L* and hue angle values, while a* and chroma values increased. The addition of extracts has been shown to result in comparable color changes in fruit juices in a number of studies (Altunkaya et al. 2013; Muzolf‐Panek and Waśkiewicz 2022). In general, dark colors are associated with higher total phenolic content (Fischer et al. 2011). Muzolf‐Panek and Waśkiewicz (2022) observed that a* values of red grape peels were related to DPPH antioxidant activity and total phenolic content. Indeed, the addition of extract to PJs resulted in a decrease in the hue angle values of the samples, which approached 0°. This indicates that the samples exhibited a red color. Conversely, the SPJ sample that added the extract in the highest ratio had lower a* values compared to the ESPJ and ESHPJ samples despite its higher anthocyanin content and DPPH activity. This contradiction may be related to the phenolic and anthocyanin composition of the S extract. Furthermore, it may be explained that the addition of the S extract may have caused less redness in the color of the PJ in comparison to the ES and ESH extracts due to the phenolic‐anthocyanin copigmentation.

**TABLE 2:** The color values of pomegranate juice (PJ) samples according to Duncan Multiple Comparison Test results.

Variation sources	*n*	L*	a*	b*	Hue angle	Chroma
PJ sample						
Control	20	61.71 ± 1.39^a^	14.41 ± 1.74^e^	38.12 ± 2.14^b^	67.78 ± 3.21^a^	41.98 ± 1.51^c^
SPJ	20	54.01 ± 1.53^d^	18.76 ± 2.45^b^	39.7 ± 1.64^a^	64.36 ± 3.38^cd^	45.52 ± 1.09^a^
HPJ	20	60.19 ± 1.64^b^	17.47 ± 2.12^c^	38.63 ± 1.83^b^	65.07 ± 3.22^c^	43.80 ± 1.25^b^
ESPJ	20	54.11 ± 1.24^d^	19.26 ± 2.11^ab^	38.17 ± 1.29^b^	63.11 ± 3.03^e^	43.98 ± 0.71^b^
TSPJ	20	59.01 ± 1.19^c^	15.74 ± 1.59^d^	38.46 ± 1.76^b^	66.47 ± 2.82^b^	42.56 ± 1.10^c^
ESHPJ	20	54.38 ± 1.60^d^	19.77 ± 2.31^a^	39.68 ± 1.48^a^	63.39 ± 3.20^de^	45.79 ± 0.87^a^
Temperature (°C)						
4	60	56.65 ± 0.88^b^	20.52 ± 1.01^a^	34.36 ± 0.38^b^	59.73 ± 1.39^b^	40.75 ± 0.44^b^
25	60	57.82 ± 0.94^a^	14.62 ± 1.25^b^	43.23 ± 1.04^a^	70.32 ± 1.90^a^	47.12 ± 0.58^a^
Storage time (Day)						
0	24	49.18 ± 0.86^e^	31.48 ± 0.93^a^	30.10 ± 0.56^e^	43.93 ± 0.59^e^	43.61 ± 1.00^bc^
15	24	54.16 ± 0.97^d^	20.46 ± 1.07^b^	36.46 ± 0.79^d^	60.73 ± 1.57^d^	42.21 ± 0.63^d^
30	24	57.80 ± 1.02^c^	15.91 ± 1.00^c^	40.10 ± 1.03^c^	67.99 ± 1.61^c^	43.56 ± 0.75^c^
45	24	61.19 ± 0.99^b^	11.13 ± 0.82^d^	42.46 ± 1.34^b^	74.64 ± 1.43^b^	44.21 ± 1.13^b^
60	24	63.84 ± 1.13^a^	8.85 ± 0.93^e^	44.84 ± 1.62^a^	77.86 ± 1.53^a^	46.09 ± 1.42^a^

*Note:* The data indicates a statistically significant difference between the means represented by different letters within the same column (*p* ≤ 0.05). SPJ: The PJ containing extract obtained from solvent extraction. HPJ: The PJ containing the extract obtained from homogenizer‐assisted extraction. ESPJ: The PJ containing extract obtained from enzymatic and solvent‐assisted extraction. TSPJ: The PJ containing the extract obtained from total liquefaction and solvent‐assisted extraction. ESHPJ: The PJ containing extract obtained from enzymatic, solvent, and homogenizer‐assisted extraction.

The elevation of the storage temperature of PJs was found to result in an increase in L*, b*, hue angle, and chroma values, while a decrease was observed in a* values. Similarly, Oziyci et al. (2013) and Qu et al. (2013) observed an increase in L* and b* values and a decrease in a* values in PJs and pomegranate peel liquid extracts with an increase in temperature. In one study, the decrease in the a* value of tannin‐added turnip juice because of an increased storage temperature was attributed to anthocyanin degradation (Kayapınar 2021). Given that anthocyanins are inherently unstable, their colors can undergo a transformation from orange to blue to yellow or even colorless, as a result of heat treatments. Ultimately, brown polymers are formed (Gumienna et al. 2016). In the present study, the change in color values in response to temperature can be primarily attributed to the degradation of anthocyanins.

During the storage period of PJs, an increase was observed in the L*, b*, hue angle, and chroma values, while a decrease was determined in a* values. Similar changes in L* (Trigo, Alexandre, Silva, et al. 2020), a* (Kayapınar 2021), hue angle (Garcia‐Hernandez et al. 2020) and chroma (Qu et al. 2013) values have been observed in other phenolic‐enriched juices. The chemical processes responsible for the color change of red fruit juices include polymerization, anthocyanin degradation, and non‐enzymatic browning (Garcia‐Hernandez et al. 2020). During storage, the hue angle of the samples approached 90°; therefore, an increase in yellowness was observed. This was attributed to the decrease in phenolic compounds and anthocyanins.

Figures S2–S6 show the interactions of L*, a*, b*, hue angle, and chroma color values of PJ samples. An increase in L* values was observed during the storage of samples stored at 4°C and 25°C. Among the samples stored at 4°C, the L* value of the HPJ sample demonstrated the highest increase, whereas among the samples stored at 25°C, the control sample exhibited the most significant increase (Figure S2). It is thought that the reactions occurring as the HPJ sample contains more impurities depending on the method are rapid at the beginning, but transition to a stationary phase after the 30th day. This change in the samples can be explained by the low stability of the anthocyanin content and the low level of formation of possible copigmentation. The a* value of the TSPJ sample at 4°C was found to be more successful than the other samples due to the slower rate of degradation. This can be attributed to the higher stability of the copigmented components and anthocyanin composition of the TSPJ sample at low temperatures (Figure S3). The alteration in b* values of the ESPJ and ESHPJ samples during storage at 25°C was limited in comparison to other samples. In the analogous interaction graph for the a* value, the values of these samples were found to be higher. Consequently, it can be said that the increase in b* values was constrained while the a* values were preserved due to the more copigmented properties of the samples (Figure S4). The TSPJ sample, which had initially exhibited the highest hue angle value, demonstrated lower values on the 15th day and subsequently (Figure S5). Similarly, the rate of increase in hue angle value of the ESPJ sample decreased after the 15th day and exhibited the lowest hue angle at the end of the storage. The control sample demonstrated a rapid increase in the initial 15 days of storage and was found to have the highest hue angle at the end of the storage period. Consequently, it can be posited that the color of the control sample approached yellowness, while its redness decreased. Among the samples containing enzymatically treated extracts, it was evaluated that the reaction rate of TSPJ and ESPJ samples was lower due to the fact that they contained more stable, copigmented and/or polymerized anthocyanins. During the storage period, an increase in the chroma values of the samples stored at 25°C was observed from the 15th day, while a slight decrease was observed in the samples stored at 4°C (Figure S6). The chroma value of the ESPJ sample at both temperatures was found to be very close to each other. In a study, as a result of PJ being stored at different temperatures, low color intensity was determined at 5°C due to slow anthocyanin degradation, while high color intensity (browning) was determined at 20°C and 37°C due to increased degradation (Hafizov and Hafizov 2021). It is hypothesized that the ESPJ sample may have demonstrated high color stability against temperature changes due to the high amount of copigmented anthocyanin components it contains.

### 3.4 Total Phenolic Content

Duncan Multiple Comparison Test results indicated that the total phenolic content of the PJ samples was found to be highest in the SPJ sample (6.23 mg GAE/mL) and the lowest in the control sample (4.25 mg GAE/mL) (Table 3). As anticipated, the addition of pomegranate by‐product extract led to a notable elevation (between 30.1% and 46.6% compared to control) in the total phenolic content of the samples. As described in the methods section, the extracts were added to PJs according to the sensory test. The extract obtained by solvent extraction could be added in a higher proportion due to its low phenolic content. Therefore, the SPJ sample was evaluated to have higher phenolic content compared to other samples.

**TABLE 3:** The total phenolic, total flavonoid, and hydrolyzable tannin values of pomegranate juice (PJ) samples according to Duncan Multiple Comparison Test results.

Variation sources	*n*	Total phenolic content (mg GAE/mL)	Total flavonoid content (mg RE/mL)	Hydrolyzable tannin content (mg TAE/mL)
PJ sample				
Control	20	4.25 ± 0.08^e^	2.51 ± 0.06^e^	7.23 ± 0.16^e^
SPJ	20	6.23 ± 0.11^a^	3.75 ± 0.05^a^	11.50 ± 0.29^a^
HPJ	20	5.87 ± 0.09^b^	3.62 ± 0.07^b^	10.88 ± 0.14^b^
ESPJ	20	5.78 ± 0.10^c^	3.48 ± 0.05^cd^	10.61 ± 0.17^c^
TSPJ	20	5.53 ± 0.11^d^	3.45 ± 0.06^d^	10.21 ± 0.19^d^
ESHPJ	20	5.82 ± 0.07^c^	3.49 ± 0.06^c^	10.57 ± 0.11^c^
Temperature (°C)				
4	60	5.66 ± 0.01^a^	3.45 ± 0.06^a^	10.29 ± 0.03^a^
25	60	5.50 ± 0.01^b^	3.32 ± 0.06^b^	10.05 ± 0.03^b^
Storage time (Day)				
0	24	5.97 ± 0.14^a^	3.76 ± 0.09^a^	11.09 ± 0.31^a^
15	24	5.88 ± 0.14^b^	3.51 ± 0.10^b^	10.81 ± 0.31^b^
30	24	5.78 ± 0.14^c^	3.31 ± 0.09^c^	9.99 ± 0.28^c^
45	24	5.22 ± 0.13^d^	3.21 ± 0.08^d^	9.55 ± 0.30^d^
60	24	5.04 ± 0.13^e^	3.12 ± 0.08^e^	9.39 ± 0.30^e^

*Note:* The data indicates a statistically significant difference between the means represented by different letters within the same column (*p* ≤ 0.05). SPJ: The PJ containing extract obtained from solvent extraction. HPJ: The PJ containing the extract obtained from homogenizer‐assisted extraction. ESPJ: The PJ containing extract obtained from enzymatic and solvent‐assisted extraction. TSPJ: The PJ containing the extract obtained from total liquefaction and solvent‐assisted extraction. ESHPJ: The PJ containing extract obtained from enzymatic, solvent, and homogenizer‐assisted extraction.

A review of the literature reveals that the total phenolic content of various PJs ranges from 0.78 to 9.47 mg/mL (Gözlekçi et al. 2011; Hmid et al. 2017). The total phenolic content of PJ is reported to be dependent on a number of factors, including the variety, the harvesting method, storage, production method, and clarification (Hegazi et al. 2021). Shahkoomahally et al. (2021) reported that the total phenolic content of pomegranate peel extracts obtained by the traditional extraction ranged from 365.71 to 1167.40 mg/L, while the phenolic content of PJ was between 1313.08 and 1700.07 mg/L. A study demonstrated that the total phenolic content of PJ increased by 47% when 50 g/L of pomegranate albedo homogenate was added (Vázquez‐Araújo et al. 2011). Similarly, the total phenolic content of carrot juice was found to increase by a factor of 3.2 when pomegranate peel extract (5 mg/mL) was added, in comparison to the control sample (Trigo, Alexandre, Silva, et al. 2020).

The total phenolic content of the samples was observed to decline during the storage period. The total phenolic content was found to be adversely affected by both elevated storage temperature and prolonged storage time, with an average loss of 15.6% observed at the end of the storage period (Table 3). Phenolic substances are known to decrease during storage due to reactions such as oxidation and polymerization (Garcia‐Hernandez et al. 2020; Qu et al. 2013). The decrease in the phenolic compounds in PJ (pulsed light and thermally pasteurized) stored in the refrigerator (4°C) for 50 days was explained by the formation of complex polymeric structures by proteins and their precipitates (Pravallika et al. 2023). In this regard, the nano‐delivery system has the potential to enhance health benefits and improve nutritional value (Andishmand, Azadmard‐Damirchi, et al. 2023). In the study conducted by Zahed et al. (2023), phenolic extracts obtained from pomegranate peel using different methods were nanocapsulated by diverse coating materials.

The two‐ and three‐way interactions of the total phenolic content of the PJ samples were demonstrated in Figure S7. The total phenolic content of the HPJ, ESPJ, and TSPJ samples was found to be similar at both temperatures and did not show any significant change over the 30‐day period. This stability can be attributed to the fact that co‐pigmentation in the samples provides stability against temperature changes and preserves the phenolic content (Pravallika et al. 2023). In this case, the composition and quantity of phenolic components, as well as the quantity of co‐pigmented components, are effective. Furthermore, this stability may also be associated with the release of low molecular weight phenolic compounds (ellagic acid, gallic acid, punicalin) resulting from storage‐associated degradation of the hydrolyzable tannin content of the samples. A study revealed that following storage of pomegranate juices, there was an overall decrease in the punicalagin isomer content of the ellagitannin group (Mena et al. 2013).

According to Vrhovsek et al. (2004), the recommended daily intake of polyphenols is 1 g/day. The quantity of sample required to provide the aforementioned intake was determined as 236.5 mL for the control, 161.3 mL for the SPJ, 171.2 mL for the HPJ, 173.9 mL for the ESPJ, 181.7 mL for the TSPJ, and 172.7 mL for the ESHPJ. Furthermore, apple and other fruit juices can be used to further increase the nutrition of PJ, which is rich in phenolic compounds.

### 3.5 Total Flavonoid Content

The highest total flavonoid content for Duncan Multiple Comparison Test results was observed in the SPJ sample, while the control sample exhibited the lowest content (Table 3). The incorporation of pomegranate by‐product extract resulted in a statistically significant increase in total flavonoid content (27.4%–49.4%), in comparison to the control sample. The observed increase in total flavonoid content among the samples with the addition of extracts was consistent with the observed change in total phenolic content. It has been documented that the total flavonoid content of pomegranate peel extract is significantly higher than that of PJ (Hegazi et al. 2021; Shahkoomahally et al. 2021). The addition of pomegranate peel extract (5 mg/mL) to carrot juice resulted in a 7‐fold increase in the total flavonoid content of the product, in comparison to the control sample (Trigo, Alexandre, Silva, et al. 2020). The present study demonstrated that the flavonoid increase in the samples to which the enzymatic‐treated extract had been added was less pronounced than in the other samples. This was attributed to the degradation of flavonoid structures in the samples due to enzymatic activity.

The total flavonoid content of the samples exhibited a decline with an increase in storage temperature (Table 3). The stability of flavonoids is dependent on temperature, light, and oxygen (Zheng et al. 2011). A similar study demonstrated that the total flavonoid content of watermelon juice was diminished at elevated storage temperatures due to structural changes (Mohamad Salin et al. 2022). In contrast, some studies have indicated that flavonoid content may be increased as a result of high storage temperatures. In one study, the rise in flavonoid concentration observed in PJs following thermal processing was attributed to the degradation of polymeric phenolics (Mena et al. 2013). The discrepancies between the studies were determined to be attributable to variations in temperature during the heat treatment and storage processes.

The total flavonoid content of PJs was observed to decline during the storage period (Table 3). As similar to other polyphenols, flavonoids are susceptible to degradation under processing and storage conditions (Zheng et al. 2011). Similarly, rutin, the most prevalent flavonoid in pomegranate peel, was demonstrated to diminish by 65% over a 4‐month period of cold storage (Mphahlele et al. 2017).

The two‐ and three‐way interactions of the total flavonoid content of the PJ samples were demonstrated in Figure S8. The SPJ sample with the highest total flavonoid content among the samples was quite stable during storage at both temperatures. Therefore, its flavonoid content was judged to be more suitable and stable than that of the other samples with anthocyanins. Nowicka et al. (2016) reported that the addition of flowering quince juice to sour cherry puree smoothies limited anthocyanin changes during storage. This may be due to copigmentation with proanthocyanidins. In the HPJ sample, a rapid decrease in total flavonoid content was observed at 4°C, particularly from the 15th day. At the conclusion of the storage period, the value of the sample stored at 4°C was comparable to that stored at 25°C. In a study, the mean degradation of each anthocyanin of the unprocessed and pasteurized pomegranate juices was between 23.0% and 83.0% during 10 days at 4°C. The findings of this study indicated that the stability of cyanidin was higher than that of delphinidin and pelargonidin, and that the most unstable anthocyanin was delphinidin during storage (Alighourchi et al. 2008). It can therefore be attributed that the flavonoids found in the HPJ sample are susceptible to storage even during cold storage.

### 3.6 Hydrolyzable Tannin Content

Duncan Multiple Comparison Test results of the hydrolyzable tannin content of the samples exhibited a range of 7.23 to 11.50 mg TAE/mL (Table 3). The addition of pomegranate by‐product extract resulted in a significant increase in the hydrolyzable tannin content of the samples, with the greatest increase observed in the SPJ (59.05%), followed by the HPJ (50.48%), ESPJ (46.75%), ESHPJ (46.20%), and TSPJ (41.21%), in comparison to the control sample. The increase in hydrolyzable tannin content in the TSPJ sample was found to be the lowest among the enriched samples. The protein residue of the TS extract is higher than that of the other enzyme‐processed extracts due to the addition of a higher quantity of the enzyme. The formation of complexes between tannins (aromatic ring) and enzyme residue protein (hydrophobic regions) may be the reason for this low hydrolyzable tannin content (Wang et al. 2016). Shahkoomahally et al. (2021) reported that pomegranate peel contains elevated concentrations of punicalagin A and B in comparison to the juice. Trigo, Alexandre, Silva, et al. (2020) determined the addition of pomegranate peel extract (5 mg/mL) to carrot juice resulted in an increase in the hydrolyzable content of the product.

As a result of the elevated storage temperature and the extended storage period, a reduction in the hydrolyzable tannin content of the samples was observed (Table 3). It is widely acknowledged that technological processes or high temperatures applied during storage lead to the degradation of polyphenols (Zheng et al. 2011). In a study by Teleszko et al. (2016), it was determined that during the storage of turbid strawberry juice at 4°C and 20°C, the sample stored at 20°C exhibited a higher free ellagic acid concentration due to the hydrolysis of ellagitannins.

Figure S9 shows the interactions of the hydrolyzable tannin content of PJ samples. The hydrolyzable tannin content in the control and the ESHPJ samples was found to be independent of temperature, while cold storage resulted in a lower content in the TSPJ sample. The phenomenon may be related to the high stability of the ESHPJ. In one study, the very stable ellagitannin content of frozen blackberries during storage was associated with a negligible degree of degradation or depolymerisation of ellagitannins (Hager et al. 2010). In the present study, protein residue was higher due to the higher amount of enzyme applied to the TS extract in comparison to other enzyme‐treated extracts. This phenomenon may be associated with the decrease in tannin content in the PJ due to the possibility of hydrolyzable tannin‐protein complex formation (Wang et al. 2016). The decline in the hydrolyzable tannin content of SPJ, HPJ, and ESPJ samples due to the increase in storage temperature can be explained by the low stability of tannins in their content.

### 3.7 The Total Monomeric Anthocyanin Content

The PJ samples were found to contain the total monomeric anthocyanin with the highest (30.31 mg C3G/L) concentration observed in the SPJ, and the lowest (24.58 mg C3G/L) in the control (Table 4). The elevated anthocyanin content of the SPJ sample was postulated to be attributable to the greater quantity of extract incorporated into this sample. In the current study, the addition of extracts to pomegranate samples resulted in an increase in the total anthocyanin content of the samples. However, this increase was found to be more limited than that of phenolic, flavonoid, and tannins. de Beer et al. (2014) determined that the addition of red plum shell extract to red plum nectar causes an increase in anthocyanin content.

**TABLE 4:** The total monomeric anthocyanin content and antioxidant activity values of pomegranate juice (PJ) samples according to Duncan Multiple Comparison Test results.

Variation sources	*n*	Total monomeric anthocyanin content (mg C3G/L)	DPPH antioxidant activity (mg TEAA/mL)
PJ sample			
Control	20	24.58 ± 4.37^d^	16.25 ± 0.62^e^
SPJ	20	30.31 ± 5.16^a^	30.76 ± 0.93^a^
HPJ	20	27.94 ± 4.79^b^	27.29 ± 0.63^b^
ESPJ	20	27.02 ± 4.63^b^	25.93 ± 0.68^d^
TSPJ	20	27.23 ± 4.64^bc^	26.77 ± 0.70^c^
ESHPJ	20	27.93 ± 4.83^c^	27.72 ± 0.82^b^
Temperature (°C)			
4	60	39.81 ± 1.37^a^	26.50 ± 0.65^a^
25	60	15.19 ± 2.75^b^	25.08 ± 0.78^b^
Storage time (Day)			
0	24	55.06 ± 0.69^a^	29.40 ± 1.04^a^
15	24	32.83 ± 3.16^b^	28.04 ± 1.06^b^
30	24	19.59 ± 3.51^c^	26.03 ± 0.99^c^
45	24	16.49 ± 3.46^d^	24.45 ± 0.97^d^
60	24	13.55 ± 2.84^e^	21.02 ± 0.85^e^

*Note:* The data indicates a statistically significant difference between the means represented by different letters within the same column (*p* ≤ 0.05). SPJ: The PJ containing extract obtained from solvent extraction. HPJ: The PJ containing the extract obtained from homogenizer‐assisted extraction. ESPJ: The PJ containing extract obtained from enzymatic and solvent‐assisted extraction. TSPJ: The PJ containing the extract obtained from total liquefaction and solvent‐assisted extraction. ESHPJ: The PJ containing extract obtained from enzymatic, solvent, and homogenizer‐assisted extraction.

Duncan Multiple Comparison Test results showed that the total monomeric anthocyanin content of the PJ was observed to decline in conjunction with an increase in the storage temperature and duration (Table 4). The mean reduction was 75% at the end of the 60‐day storage period. A comparable decline in anthocyanin was observed in different juices (Kayapınar 2021; Pravallika et al. 2023). The degradation of anthocyanins is accelerated by storage at elevated temperatures and under light. Consequently, the formation of colorless or undesirable brownish compounds may occur (Pravallika et al. 2023). The loss of anthocyanins, which are unstable compounds, may be attributed to a number of chemical reactions, including condensation, oxidation, and polymerization reactions (Garcia‐Hernandez et al. 2020). It was therefore stated that the nanoencapsulation of pomegranate peel extract can be useful in the formulation of functional foods due to its acceptable taste and ensuring high stability during storage (Andishmand, Azadmard‐Damirchi, et al. 2023). Zahed et al. (2023) investigated the nanoencapsulation of anthocyanin extracts obtained from pomegranate peel using different extraction methods.

The two‐ and three‐way interactions of the total anthocyanin content of the PJ samples were demonstrated in Figure S10. The anthocyanin values of the samples stored at 25°C were found to be closer to each other than those stored at 4°C, and this value decreased to zero on the 45th day. Mena et al. (2013) determined that 90% of anthocyanins were degraded when stored between 77 and 233 days at 5°C and 8 to 15 days at 25°C. The control sample exhibited the lowest total monomeric anthocyanin content up to Day 30 at both temperatures. de Beer et al. (2014) stored red plum nectar containing red plum peel extract for a period of 3 months. It was determined that the total anthocyanin content of the enriched sample after storage was higher than that of the control sample. This phenomenon was associated with copigmentation between phenolic components. In the present study, the degradation of anthocyanins was evaluated to be oxidation‐based.

### 3.8 DPPH Radical Scavenging Antioxidant Activity

Duncan Multiple Comparison Test results for the antioxidant activity of the PJ samples were found to be 16.25 and 30.76 mg TEAA/mL (Table 4). The incorporation of pomegranate by‐product extract into PJs led to a substantial increase (59.6%–89.3%) in the antioxidant activity content of the samples. As expected, a strong correlation was determined between antioxidant activity and total phenolic content (0.968), total flavonoid content (0.970) and hydrolyzable tannin content (0.969). Similarly, Andishmand, Masoumi, et al. (2023) also reported a strong correlation between the total phenolic content of pomegranate peel (extracted using ultrasonication and dynamic maceration‐assisted extraction) and their DPPH radical scavenging activity. A number of studies have indicated that the addition of pomegranate peel extract to fruit juices may result in an increase in the antioxidant capacity of the samples (Barros et al. 2014; Trigo, Alexandre, Oliveira, et al. 2020). The antioxidant activity of PJ can be attributed primarily to the presence of hydrolyzable tannins (ellagitannins and gallotannins), ellagic acid, anthocyanins (cyanidin, delphinidin and pelargonidin glycosides) and other flavonoid compounds (quercetin, kaempferol and luteolin glycosides) derived from the pomegranate peel. The results of the present study were found to be consistent with those reported in the existing literature.

The antioxidant activity of PJs was observed to decline with increasing storage temperature and duration. Following a storage period of 60 days, the antioxidant capacity exhibited a mean reduction of 28.5% (Table 4). This reduction is consistent with the observed decline in total phenolic, total flavonoid, and hydrolyzable tannin contents of the samples. Comparable reductions were observed in the radical scavenging activity of carrot juice with pomegranate peel extract (Trigo, Alexandre, Oliveira, et al. 2020) and a smoothie containing beet leaf extract (Fernandez et al. 2020).

The present study revealed that the antioxidant activity values of SPJ samples stored at varying temperatures were found to be almost identical, while significant differences were observed in other samples (Figure S11). While no significant change was observed in the antioxidant activity of HPJ and TSPJ samples during the initial 30 days, a marked decrease was observed in the ongoing period. In other samples, the decrease occurred gradually during storage. The observation that the total phenolic content of HPJ and TSPJ samples did not show any significant change over 30 days was found to be consistent with this result. The presence of copigmentation in the samples was attributed to be related to this stability in the phenolic content. The strong correlation between the total phenolic content and DPPH radical scavenging activity (Andishmand, Masoumi, et al. 2023) indicates that the stability of HPJ and TSPJ samples may be due to copigmentation.

### 3.9 Sensorial Properties

Duncan Multiple Comparison Test results indicated that the sensory analysis of the PJs was presented in Table 5. The control sample was rated the highest for appearance. The addition of extract to PJ resulted in a reduction in the appearance scores. The turbidity analysis clearly showed that the addition of the extract increased the turbidity of the samples. The decrease in appearance scores was caused by turbidity. Similarly, Dalabasmaz (2013) determined that the addition of echinacea extract to apple juice and apricot nectar resulted in a decrease in appearance scores compared to control samples. The ESPJ and control samples had the highest color scores, while the ESHPJ samples had the lowest. It was evaluated that this decrease in color score in most of the extract‐added PJs is associated with the turbidity of the sample. The ESPJ sample had the best odor score, while the TSPJ sample had the worst. Dalabasmaz (2013) demonstrated that the odor scores of the samples declined in comparison to the control samples following the addition of echinacea extract to apple juice and apricot nectar. The authors proposed that the specific aromatic compounds in the juice were suppressed by the extract. In the present study, an extract from the same origin as the juice was used, thereby limiting the effect of the extract on the odor. The viscosity scores of the samples were found to be statistically similar in all samples (*p* > 0.05).

**TABLE 5:** The sensorial properties of pomegranate juice (PJ) samples according to the Duncan Multiple Comparison Test results.

Variation sources	*n*	Appearance	Color	Odor	Viscosity	Taste	Aftertaste	Overall acceptability
PJ sample								
Control	20	5.61 ± 0.57^a^	5.35 ± 0.61^a^	6.00 ± 0.54^bc^	7.84 ± 0.12^a^	4.37 ± 0.47^c^	4.34 ± 0.43^c^	5.15 ± 0.50^cd^
SPJ	20	5.24 ± 0.60^b^	5.18 ± 0.59^ab^	6.08 ± 0.51^ab^	7.79 ± 0.16^a^	4.11 ± 0.46^c^	3.40 ± 0.41^e^	5.01 ± 0.53^d^
HPJ	20	5.40 ± 0.57^ab^	5.23 ± 0.60^ab^	5.91 ± 0.58^bc^	7.83 ± 0.16^a^	4.24 ± 0.40^c^	3.91 ± 0.38^d^	4.90 ± 0.46^d^
ESPJ	20	5.20 ± 0.55^b^	5.36 ± 0.56^a^	6.33 ± 0.53^a^	7.73 ± 0.19^a^	5.65 ± 0.54^a^	5.40 ± 0.52^a^	5.73 ± 0.54^a^
TSPJ	20	4.93 ± 0.62^c^	5.19 ± 0.60^ab^	5.77 ± 0.61^c^	7.63 ± 0.17^a^	4.84 ± 0.46^b^	4.11 ± 0.31^cd^	5.58 ± 0.45^ab^
ESHPJ	20	4.76 ± 0.60^c^	5.04 ± 0.58^b^	5.83 ± 0.64^bc^	7.77 ± 0.16^a^	4.88 ± 0.47^b^	4.74 ± 0.42^b^	5.35 ± 0.47^bc^
Temperature (°C)								
4	60	6.41 ± 0.22^a^	6.33 ± 0.24^a^	6.95 ± 0.18^a^	7.91 ± 0.08^a^	5.52 ± 0.19^a^	4.99 ± 0.18^a^	6.19 ± 0.17^a^
25	60	3.97 ± 0.35^b^	4.12 ± 0.36^b^	5.02 ± 0.38^b^	7.62 ± 0.10^b^	3.84 ± 0.29^b^	3.65 ± 0.28^b^	4.38 ± 0.32^b^
Storage time (Day)								
0	24	8.51 ± 0.07^a^	8.45 ± 0.07^a^	8.50 ± 0.06^a^	8.51 ± 0.01^a^	6.96 ± 0.16^a^	6.32 ± 0.16^a^	7.49 ± 0.10^a^
15	24	6.51 ± 0.28^b^	6.88 ± 0.24^b^	8.01 ± 0.09^b^	8.37 ± 0.06^a^	6.00 ± 0.17^b^	5.73 ± 0.18^b^	6.94 ± 0.11^b^
30	24	4.79 ± 0.38^c^	4.92 ± 0.39^c^	6.11 ± 0.32^c^	7.74 ± 0.09^b^	4.25 ± 0.36^c^	3.83 ± 0.32^c^	4.99 ± 0.33^c^
45	24	3.32 ± 0.33^d^	3.24 ± 0.28^d^	4.11 ± 0.40^d^	7.23 ± 0.09^c^	3.61 ± 0.31^d^	3.30 ± 0.29^d^	4.27 ± 0.34^d^
60	24	2.82 ± 0.37^e^	2.64 ± 0.33^e^	3.20 ± 0.37^e^	6.95 ± 0.10^d^	2.57 ± 0.30^e^	2.41 ± 0.28^e^	2.74 ± 0.33^e^

*Note:* The data indicates a statistically significant difference between the means represented by different letters within the same column (*p* ≤ 0.05). SPJ: The PJ containing extract obtained from solvent extraction. HPJ: The PJ containing the extract obtained from homogenizer‐assisted extraction. ESPJ: The PJ containing extract obtained from enzymatic and solvent‐assisted extraction. TSPJ: The PJ containing the extract obtained from total liquefaction and solvent‐assisted extraction. ESHPJ: The PJ containing extract obtained from enzymatic, solvent, and homogenizer‐assisted extraction.

The ESPJ sample was identified as the most palatable of the samples tested (Table 5). The samples obtained by enzymatic extraction were found to be more palatable than the control sample. The presence of pectinase, cellulase, and xylanase enzymes was found to significantly increase the release of neutral sugars from cell walls isolated from mango mesocarp (Brito and Vaillant 2012). It has also been observed that the flavor intensity of fruit juices is enhanced during the enzymatic processes (Drider et al. 1994). This phenomenon has been linked to the utilization of enzymes such as α‐glycosidase, which facilitates the release of terpenols that are complexed with polysaccharides (Vaillant et al. 1999). Therefore, in the present study, the addition of enzymatically processed extracts to PJ resulted in an enhancement of the taste profile, attributable to the presence of free sugars and aromatic compounds.

The ESPJ sample exhibited the highest aftertaste score, while the SPJ sample demonstrated the lowest score (Table 5). The low aftertaste score observed in the SPJ sample was linked to its elevated phenolic content, which contributes to the development of sensory attributes such as astringency and bitterness. It is established that tannins present in pomegranate peels form a complex with salivary proteins, resulting in the perception of astringency in the oral cavity (Andishmand, Azadmard‐Damirchi, et al. 2023). Salgado et al. (2012) reported that the sensory acceptability of commercial tomato and strawberry orange juices was adversely affected by the addition ratio of pomegranate peel extract. This was attributed to the characteristic astringent sensation imparted by the pomegranate peels, which became more pronounced with increasing extract ratio. With regard to the overall acceptability score, the ESPJ sample demonstrated the highest score among the samples, while the SPJ and HPJ samples exhibited the lowest scores (*p* > 0.05) (Table 5). The overall acceptability of the samples was found to be consistent with the taste and aftertaste scores. The PJs containing enzymatic‐processed extracts were perceived as more favorable than the other samples. The distinctive flavor of PJ masked the taste of the added extracts, thereby allowing them to be added in certain amounts without adversely affecting the overall flavor profile. A review of the literature revealed that the addition of pomegranate peel extract to fruit juices did not significantly impact the sensory properties of the juices, and that their consumability was not found to be different from that of the control (Barros et al. 2014; Mokhtar and Ibrahim 2020).

It was observed that the sensorial properties of the samples declined in accordance with the increase in storage temperature and the prolongation of the storage period (Table 5). The appearance score of samples stored at 25°C occurred at a decreasing speed, while at 4°C it showed an increasing speed in the initial 45 days and then decreased sharply (Figure S12). The ESPJ sample exhibited the lowest appearance score during storage at 4°C and the highest score at 25°C. It was established that among the samples stored at 4°C, the TSPJ sample with the highest color score was found to have the lowest color score at 25°C (Figure S13). This result was associated with the sample being less appreciated due to having the lowest a* value and the highest tone angle at 25°C. The observed decline in appearance score was determined to be attributable to the concurrent increase in increasing brix and turbidity values of the samples. The instrumentally observed yellowish‐brownish color formation in the samples stored at 25°C was found to be less preferred by consumers. It has been documented that the storage of PJ in conditions of elevated temperature and light exposure can result in accelerated degradation of anthocyanins. Consequently, the formation of colorless or brown compounds is observed (Pravallika et al. 2023).

The decline in odor scores with the increase in temperature in the samples can be associated with the slower rate of degradation of odor components at low temperatures (Table 5). The odor scores of the samples stored at 4°C were found to be similar. It was also determined that the odor characteristics of the TSPJ and ESHPJ samples were more sensitive to high temperatures (Figure S14). Monoterpenes, which are volatile components, are present in PJ in either free or glycosidic form. During the storage or processing of the PJ, the glycosidically bound aroma compounds are released (Tripathi et al. 2014). As seen in Table 5, the observed reduction in the viscosity scores of the samples during the storage period was in accordance with the findings of the study conducted by Sharma et al. (2019). This reduction has been explained by copolymerization, interactions between phenolics, degradation of colloidal particles, and protein. It has also been associated with complex formation with pectin and phenolics during storage. The ESPJ sample had the highest taste score during storage of all the samples (Figure S15). When stored at 4°C and 25°C, only the difference between the taste scores of the SPJ sample among the PJ varieties was found to be close. The taste scores of the samples at 4°C were found to be higher. Among the samples stored at 4°C, the ESPJ sample had the highest taste score, whereas, of the samples stored at 25°C, the SPJ sample had the highest. The SPJ sample at 25°C had the highest total phenolic content. Therefore, at high temperatures, the SPJ sample may have been more appreciated due to the degradation of complex flavonoids into simple compounds that are responsible for the better flavor profile (Basak and Chakraborty 2022; Pravallika et al. 2023). The most significant discrepancy between the aftertaste scores of the PJ varieties during storage at 4°C and 25°C was observed in the ESPJ sample, while the scores of the other samples were found to be close to each other (Figure S16). The variation in the overall acceptability scores of the HPJ, TSPJ, and ESPJ samples stored at different temperatures was found to be more limited and less dependent on temperature than the other samples (Figure S17). The reduction in the taste scores of the samples with the increase in temperature was attributed to the potential loss of volatile aromatic compounds during storage (Sharma et al. 2019). Furthermore, the gradual transition of the colors from red to lighter colors, coupled with a cloudy and sedimentary appearance, may have negatively affected the taste appreciation. This is evidenced by the decline in all sensorial scores with increasing storage temperature and duration, which corroborates the observed reduction in general acceptability. Consequently, research into nanoencapsulation as an alternative solution is at the forefront, with the aim of increasing the sensory acceptable levels of phenolic compounds added to food, as well as increasing their stability and bioavailability (Andishmand, Azadmard‐Damirchi, et al. 2023).

## 4 Conclusions

The objective of this study was to produce upcycled PJs enriched with phenolic compounds obtained from pomegranate by‐products through the utilization of green extraction methods. The addition of extracts to PJ samples led to an enhancement in the concentrations of phenolics, flavonoids, tannins, and anthocyanins, as well as antioxidant activity. However, the addition of extracts resulted in a detrimental impact on turbidity, L* value, and hue value. Among the PJs, the SPJ sample exhibited the highest brix and turbidity values, while the control sample demonstrated the lowest values. Statistical analysis revealed no significant differences in turbidity values among samples containing extracts processed with enzymes. The highest L* value was found in the control sample, while the highest a* value was determined in the ESHPJ sample. The incorporation of extract resulted in an increase in the total phenolic content of the samples in comparison to the control sample, with the highest increase of 46.58% in the SPJ sample and the lowest increase of 30.11% in the TSPJ sample. The highest sensory acceptance scores were observed in PJs that had been enriched using enzymatic extraction processes. Given the low addition rate to PJs (41.5% less than S extract, 25.9% less than ESH, 15.2% less than TS and 14.5% less than ES) and the brief extraction time (only 1 min), the utilization of H extract was determined to be a more optimal choice in terms of chemical, physical, and sensory characteristics, as well as economically, for the fortification of PJ. During storage at 4°C, the change in total phenolic content of the ESHPJ sample remained limited, while the hydrolyzable tannin content of the TSPJ sample showed a sharp decrease, especially after the 30th day. Among the PJ samples, the total monomeric anthocyanin content demonstrated an average decrease of 50.78% following storage at 4°C and 100% following storage at 25°C. No significant change was observed in the antioxidant activity of the HPJ and TSPJ samples during the initial 30‐day period. For storage, 4°C was found to be more favorable than 25°C as it limits physicochemical and sensory changes. The findings demonstrate that pomegranate by‐products can be further processed to produce sensorially acceptable, bioactive compound content, and antioxidant activity‐enhanced value‐added products. This is an effective strategy for sustainable food production. The evaluation of by‐products is an important step in preventing the negative effects they will create on the environment, as well as the contribution they will provide to food.

## Author Contributions

**Merve Aydin:** investigation (equal), methodology (equal), writing – original draft (equal). **Ismail Tontul:** conceptualization (equal), writing – original draft (equal). **Selman Turker:** funding acquisition (equal), resources (equal), supervision (equal), writing – review and editing (equal).

## Conflicts of Interest

The authors declare no conflicts of interest.

## Supporting information


Figures S1‐S17.

## Data Availability

Data will be made available on request.
