# Supplementary material for: Upcycling of Pomegranate By‐Products: Pomegranate Juice Enrichment With Phenolics‐Rich Pomegranate By‐Product Extracts Obtained by Green Extraction Methods
Source: Food Sci Nutr. 2025 May 29;13(6):e70250. doi: 10.1002/fsn3.70250 (PMC12121441; doi:10.1002/fsn3.70250)
Supplement: Supplementary file 1 — Figures S1‐S17. [file FSN3-13-e70250-s001.docx]

**Supplementary material**

**Upcycling of Pomegranate By-Products: Pomegranate Juice Enrichment with Phenolics-Rich Pomegranate By-Product Extracts Obtained by Green Extraction Methods**

**Merve Aydin, Ismail Tontul, Selman Turker**

Necmettin Erbakan University, Faculty of Engineering, Department of Food Engineering, Konya, 42090, Türkiye

Corresponding author: Ismail Tontul

itontul@erbakan.edu.tr

**FIGURE S1**. The interaction between temperature and storage period on total soluble solid content (brix) of pomegranate juice (PJ) samples (**p≤0.01).

1. **b)**

**c) d)**

**FIGURE S2**. The interaction between temperature and storage period (a) (**p≤0.01), temperature and pomegranate juice (PJ) variety (b) (**p≤0.01), storage period and PJ variety (c) (*p≤0.05), temperature, storage period and PJ variety (d) (**p≤0.01) on L* colour values ​​of PJ samples. **SPJ**: The PJ containing extract obtained from solvent extraction, **HPJ**: The PJ containing the extract obtained from homogeniser-assisted extraction, **ESPJ**: The PJ containing extract obtained from enzymatic and solvent-assisted extraction, **TSPJ**: The PJ containing the extract obtained from total liquefaction and solvent-assisted extraction, **ESHPJ**: The PJ containing extract obtained from enzymatic, solvent and homogenizer-assisted extraction.

1. **b)**

**c) d)**

**FIGURE S3**. The interaction between temperature and storage period (a) (**p≤0.01), temperature and pomegranate juice (PJ) variety (b) (**p≤0.01), storage period and PJ variety (c) (**p≤0.01), temperature, storage period and PJ variety (d) (**p≤0.01) on a* colour values of PJ samples. **HPJ**: The PJ containing the extract obtained from homogeniser-assisted extraction, **ESPJ**: The PJ containing extract obtained from enzymatic and solvent-assisted extraction, **TSPJ**: The PJ containing the extract obtained from total liquefaction and solvent-assisted extraction, **ESHPJ**: The PJ containing extract obtained from enzymatic, solvent and homogenizer-assisted extraction.

**a) b)**

**c) d)**

**FIGURE S4**. The interaction between temperature and storage period (a) (**p≤0.01), temperature and pomegranate juice (PJ) variety (b) (**p≤0.01), storage period and PJ variety (c) (**p≤0.01), temperature, storage period and PJ variety (d) (*p≤0.05) on b* colour values of PJ samples. **HPJ**: The PJ containing the extract obtained from homogeniser-assisted extraction, **ESPJ**: The PJ containing extract obtained from enzymatic and solvent-assisted extraction, **TSPJ**: The PJ containing the extract obtained from total liquefaction and solvent-assisted extraction, **ESHPJ**: The PJ containing extract obtained from enzymatic, solvent and homogenizer-assisted extraction.

**a) b)**

**c) d)**

**FIGURE S5**. The interaction between temperature and storage period (a) (**p≤0.01), temperature and pomegranate juice (PJ) variety (b) (**p≤0.01), storage period and PJ variety (c) (**p≤0.01), temperature, storage period and PJ variety (d) (**p≤0.01) on hue angle colour values of PJ samples. **HPJ**: The PJ containing the extract obtained from homogeniser-assisted extraction, **ESPJ**: The PJ containing extract obtained from enzymatic and solvent-assisted extraction, **TSPJ**: The PJ containing the extract obtained from total liquefaction and solvent-assisted extraction, **ESHPJ**: The PJ containing extract obtained from enzymatic, solvent and homogenizer-assisted extraction.

1. **b)**

**c) d)**

**FIGURE S6**. The interaction between temperature and storage period (a) (**p≤0.01), temperature and pomegranate juice (PJ) variety (b) (**p≤0.01), storage period and PJ variety (c) (**p≤0.01), temperature, storage period and PJ variety (d) (**p≤0.01) on chroma colour values of PJ samples. **HPJ**: The PJ containing the extract obtained from homogeniser-assisted extraction, **ESPJ**: The PJ containing extract obtained from enzymatic and solvent-assisted extraction, **TSPJ**: The PJ containing the extract obtained from total liquefaction and solvent-assisted extraction, **ESHPJ**: The PJ containing extract obtained from enzymatic, solvent and homogenizer-assisted extraction.

1. **b)**

**c) d)**

**FIGURE S7.** The interaction between temperature and storage period (a) (**p≤0.01), temperature and pomegranate juice (PJ) variety (b) (**p≤0.01), storage period and PJ variety (c) (**p≤0.01), temperature, storage period and PJ variety (d) (**p≤0.01) on total phenolic content values ​​of PJ samples (**p≤0.01). **SPJ**: The PJ containing extract obtained from solvent extraction, **HPJ**: The PJ containing the extract obtained from homogeniser-assisted extraction, **ESPJ**: The PJ containing extract obtained from enzymatic and solvent-assisted extraction, **TSPJ**: The PJ containing the extract obtained from total liquefaction and solvent-assisted extraction, **ESHPJ**: The PJ containing extract obtained from enzymatic, solvent and homogenizer-assisted extraction.

**a) b)**

**c) d)**

**FIGURE S8.** The interaction between temperature and storage period (a) (**p≤0.01), temperature and pomegranate juice (PJ) variety (b) (**p≤0.01), storage period and PJ variety (c) (**p≤0.01), temperature, storage period and PJ variety (d) (**p≤0.01) on total flavonoid content values ​​of PJ samples. **SPJ**: The PJ containing extract obtained from solvent extraction, **HPJ**: The PJ containing the extract obtained from homogeniser-assisted extraction, **ESPJ**: The PJ containing extract obtained from enzymatic and solvent-assisted extraction, **TSPJ**: The PJ containing the extract obtained from total liquefaction and solvent-assisted extraction, **ESHPJ**: The PJ containing extract obtained from enzymatic, solvent and homogenizer-assisted extraction.

**a) b)**

**c) d)**

**FIGURE S9.** The interaction between temperature and storage period (a) (**p≤0.01), temperature and pomegranate juice (PJ) variety (b) (**p≤0.01), storage period and PJ variety (c) (**p≤0.01), temperature, storage period and PJ variety (d) (**p≤0.01) on hydrolysable tannin content values ​​of PJ samples. **SPJ**: The PJ containing extract obtained from solvent extraction, **HPJ**: The PJ containing the extract obtained from homogeniser-assisted extraction, **ESPJ**: The PJ containing extract obtained from enzymatic and solvent-assisted extraction, **TSPJ**: The PJ containing the extract obtained from total liquefaction and solvent-assisted extraction, **ESHPJ**: The PJ containing extract obtained from enzymatic, solvent and homogenizer-assisted extraction.

**a) b)**

**c) d)**

**FIGURE S10.** The interaction between temperature and storage period (a) (**p≤0.01), temperature and pomegranate juice (PJ) variety (b) (**p≤0.01), storage period and PJ variety (c) (**p≤0.01), temperature, storage period and PJ variety (d) (**p≤0.01) on total monomeric anthocyanin content values ​​of PJ samples. **SPJ**: The PJ containing extract obtained from solvent extraction, **HPJ**: The PJ containing the extract obtained from homogeniser-assisted extraction, **ESPJ**: The PJ containing extract obtained from enzymatic and solvent-assisted extraction, **TSPJ**: The PJ containing the extract obtained from total liquefaction and solvent-assisted extraction, **ESHPJ**: The PJ containing extract obtained from enzymatic, solvent and homogenizer-assisted extraction.

**a) b)**

**c)**

**FIGURE S11.** The interaction between temperature and storage period (a) (**p≤0.01), temperature and pomegranate juice (PJ) variety (b) (**p≤0.01) and storage period and PJ variety (c) (**p≤0.01) on DPPH antioxidant activity values ​​of PJ samples. **SPJ**: The PJ containing extract obtained from solvent extraction, **HPJ**: The PJ containing the extract obtained from homogeniser-assisted extraction, **ESPJ**: The PJ containing extract obtained from enzymatic and solvent-assisted extraction, **TSPJ**: The PJ containing the extract obtained from total liquefaction and solvent-assisted extraction, **ESHPJ**: The PJ containing extract obtained from enzymatic, solvent and homogenizer-assisted extraction.

**a) b)**

**c)**

**FIGURE S12.** The interaction between temperature and storage period (a) (**p≤0.01), temperature and pomegranate juice (PJ) variety (b) (**p≤0.01) and temperature, storage period and PJ variety (c) (*p≤0.05) on appearance values ​​of PJ samples. **SPJ**: The PJ containing extract obtained from solvent extraction, **HPJ**: The PJ containing the extract obtained from homogeniser-assisted extraction, **ESPJ**: The PJ containing extract obtained from enzymatic and solvent-assisted extraction, **TSPJ**: The PJ containing the extract obtained from total liquefaction and solvent-assisted extraction, **ESHPJ**: The PJ containing extract obtained from enzymatic, solvent and homogenizer-assisted extraction.

**a) b)**

**c) d)**

**FIGURE S13.** The interaction between temperature and storage period (a) (**p≤0.01), temperature and pomegranate juice (PJ) variety (b) (**p≤0.01), storage period and PJ variety (c) (*p≤0.05), temperature, storage period and PJ variety (d) (*p≤0.05) on colour sensorial values ​​of PJ samples. **SPJ**: The PJ containing extract obtained from solvent extraction, **HPJ**: The PJ containing the extract obtained from homogeniser-assisted extraction, **ESPJ**: The PJ containing extract obtained from enzymatic and solvent-assisted extraction, **TSPJ**: The PJ containing the extract obtained from total liquefaction and solvent-assisted extraction, **ESHPJ**: The PJ containing extract obtained from enzymatic, solvent and homogenizer-assisted extraction.

**a) b)**

**c) d)**

**FIGURE S14.** The interaction between temperature and storage period (a) (**p≤0.01), temperature and pomegranate juice (PJ) variety (b) (**p≤0.01), storage period and PJ variety (c) (**p≤0.01), temperature, storage period and PJ variety (d) (**p≤0.01) on odour sensorial values ​​of PJ samples. **SPJ**: The PJ containing extract obtained from solvent extraction, **HPJ**: The PJ containing the extract obtained from homogeniser-assisted extraction, **ESPJ**: The PJ containing extract obtained from enzymatic and solvent-assisted extraction, **TSPJ**: The PJ containing the extract obtained from total liquefaction and solvent-assisted extraction, **ESHPJ**: The PJ containing extract obtained from enzymatic, solvent and homogenizer-assisted extraction.

**a) b)**

**c)**

**FIGURE S15.** The interaction between temperature and storage period (a) (**p≤0.01), temperature and pomegranate juice (PJ) variety (b) (**p≤0.01) and storage period and PJ variety (c) (*p≤0.05) on taste sensorial values ​​of PJ samples. **SPJ**: The PJ containing extract obtained from solvent extraction, **HPJ**: The PJ containing the extract obtained from homogeniser-assisted extraction, **ESPJ**: The PJ containing extract obtained from enzymatic and solvent-assisted extraction, **TSPJ**: The PJ containing the extract obtained from total liquefaction and solvent-assisted extraction, **ESHPJ**: The PJ containing extract obtained from enzymatic, solvent and homogenizer-assisted extraction.

1. **b)**

**FIGURE S16.** The interaction between temperature and storage period (a) (**p≤0.01) and temperature and pomegranate juice (PJ) variety (b) (**p≤0.01) on aftertaste sensorial values ​​of PJ samples. **SPJ**: The PJ containing extract obtained from solvent extraction, **HPJ**: The PJ containing the extract obtained from homogeniser-assisted extraction, **ESPJ**: The PJ containing extract obtained from enzymatic and solvent-assisted extraction, **TSPJ**: The PJ containing the extract obtained from total liquefaction and solvent-assisted extraction, **ESHPJ**: The PJ containing extract obtained from enzymatic, solvent and homogenizer-assisted extraction.

**a) b)**

**FIGURE S17.** The interaction between temperature and storage period (a) (**p≤0.01) and temperature and pomegranate juice (PJ) variety (b) (**p≤0.01) on overall acceptability sensorial values ​​of PJ samples. **SPJ**: The PJ containing extract obtained from solvent extraction, **HPJ**: The PJ containing the extract obtained from homogeniser-assisted extraction, **ESPJ**: The PJ containing extract obtained from enzymatic and solvent-assisted extraction, **TSPJ**: The PJ containing the extract obtained from total liquefaction and solvent-assisted extraction, **ESHPJ**: The PJ containing extract obtained from enzymatic, solvent and homogenizer-assisted extraction.
